# Mesenchymal Stem Cell 1 (*MSC1*)-Based Therapy Attenuates Tumor Growth Whereas *MSC2-*Treatment Promotes Tumor Growth and Metastasis

**DOI:** 10.1371/journal.pone.0045590

**Published:** 2012-09-20

**Authors:** Ruth S. Waterman, Sarah L. Henkle, Aline M. Betancourt

**Affiliations:** 1 Department of Anesthesiology, Ochsner Clinic Foundation, New Orleans, Louisiana, United States of America; 2 Tulane Center for Stem Cell Research and Regenerative Medicine, Tulane University School of Medicine, New Orleans, Louisiana, United States of America; 3 Department of Medicine, Tulane University School of Medicine, New Orleans, Louisiana, United States of America; Children's Hospital Boston/Harvard Medical School, United States of America

## Abstract

**Background:**

Currently, there are many promising clinical trials using mesenchymal stem cells (MSCs) in cell-based therapies of numerous diseases. Increasingly, however, there is a concern over the use of MSCs because they home to tumors and can support tumor growth and metastasis. For instance, we established that MSCs in the ovarian tumor microenvironment promoted tumor growth and favored angiogenesis. In parallel studies, we also developed a new approach to induce the conventional mixed pool of MSCs into two uniform but distinct phenotypes we termed *MSC1* and *MSC2*.

**Methodology/Principal Findings:**

Here we tested the *in vitro* and *in vivo* stability of *MSC1* and *MSC2* phenotypes as well as their effects on tumor growth and spread. *In vitro* co-culture of *MSC1* with various cancer cells diminished growth in colony forming units and tumor spheroid assays, while conventional MSCs or *MSC2* co-culture had the opposite effect in these assays. Co-culture of *MSC1* and cancer cells also distinctly affected their migration and invasion potential when compared to MSCs or *MSC2* treated samples. The expression of bioactive molecules also differed dramatically among these samples. *MSC1*-based treatment of established tumors in an immune competent model attenuated tumor growth and metastasis in contrast to MSCs- and *MSC2*-treated animals in which tumor growth and spread was increased. Also, in contrast to these groups, *MSC1*-therapy led to less ascites accumulation, increased CD45+leukocytes, decreased collagen deposition, and mast cell degranulation.

**Conclusion/Significance:**

These observations indicate that the *MSC1* and *MSC2* phenotypes may be convenient tools for the discovery of critical components of the tumor stroma. The continued investigation of these cells may help ensure that cell based-therapy is used safely and effectively in human disease.

## Introduction

Mesenchymal stem cells (MSCs, more accurately termed multipotent mesenchymal stromal cells) are increasingly being used in cell-based therapies of diseases ranging widely from graft-versus-host to joint and cartilage disorders [1], [2]. There are many features that make these cells attractive and practical for use in human therapy. First, MSCs are easily obtained from various adult-derived tissues, quickly expanded, and stored *ex vivo* without significant impact to their capabilities. Second, once reintroduced, MSCs preferentially home to sites of injury or inflammation and support healing and repair mostly through the local secretion of bioactive factors and modulation of immune cells. Third, MSCs from non-self (allogeneic) or self (autologous) donors can be used safely since they do not elicit harmful immune responses within the recipient host. Lastly, pre-clinical studies have demonstrated efficacy with MSCs genetically engineered to carry various therapeutics that reached their target with significant treatment benefit even in the xenogeneic setting (human cells to mouse host) (recently reviewed [3]–[5]).

Despite these promising features, there is a growing concern over the clinical use of MSCs since they are also known to home to tumors and once resident in the tumor microenvironment (TME) to support tumor growth and spread [4]–[8]. Conversely, other studies have reported that MSCs found in the TME diminish tumor growth, which has further generated some controversy in this field (reviewed in [4], [5]). Other noted concerns in the clinical use of MSCs, is the fact that we still do not have a general consensus of what defines them, and furthermore although one of their most profound clinical effects upon intravenous administration is the modulation of host immune responses, we do not yet truly understand all of their consequences upon introduction into the host [1], [9], [10]. Either way, as a result of the established clinical properties of MSC and their added propensity for the TME, modified MSCs that can act as “Trojan horses” and deliver anti-cancer therapeutics into the tumor stroma are being evaluated as a promising new targeted cell-based therapy for cancer [4], [5].

MSCs targeted to cancers are expected to contribute many soluble factors such as mitogens, extracellular matrix (ECM) proteins, angiogenic, and inflammatory factors, as well as exosomes or microvescicles, once resident in the TME [3]–[5]. MSCs are also expected to affect tumor-associated leukocytes either directly by cell-cell contact or indirectly by the secretion of trophic factors [3]–[5]. MSCs are known to affect the proliferation and differentiation of dendritic cells, monocytes/macrophages, B and T cells, NK cells, and even mast cells [3]–[5]. Many reasons have been advanced to explain the contradictory MSC role in cancer including but not limited to the heterogeneity of MSC preparations, the age or health of the MSC donor, and the experimental model or condition [3]–[5].

Our group established that MSCs in the ovarian tumor microenvironment promoted tumor growth and favored angiogenesis [7], [11], [12]. We also developed new methodology to induce the conventional mixed pool of MSCs into two uniform but distinct phenotypes, *MSC1* and *MSC2* [13]. These phenotypes were recently and successfully tested in the therapy of a mouse model of painful diabetic peripheral neuropathy [14]. This study also demonstrates the stability of these newly defined phenotypes in cell-based treatment of an immune competent disease model. We initially based their classification on several parallel observations reported within the monocyte literature. Like MSCs, heterogeneous bone marrow-derived monocytes respond to stress or “danger“ inflammatory signals and home to tissue injury. Monocyte polarization into the classically activated pro-inflammatory macrophages (M1) occurs early on in tissue repair, whereas monocyte polarization into alternatively activated macrophages (M2) follows later to help in tissue injury resolution [15], [16]. Although, this is a very simplified view of what occurs in the complex process of wound healing and repair, it provides a convenient paradigm to begin to dissect critical components within this complex biological process [17]–[19].

In this study, we similarly took advantage of this convenient paradigm in MSCs as a way to potentially resolve some of the controversy surrounding the complex role of MSCs in cancer. Indeed, *MSC1* and *MSC2* were found to have divergent effects on cancer growth and metastasis by *in vitro* and *in vivo* methods. In our experiments, *MSC1* primarily had an anti-tumor effect, whereas *MSC2* promoted tumor growth and metastases. We suggest that further investigation of these cells may provide some guidance in designing safer and more efficacious MSC-based therapies.

## Results

### *MSC1* do not Support *in vitro* Tumor Cell Growth Whereas *MSC2* Favor Tumor Cell Growth

**Figure 1:**
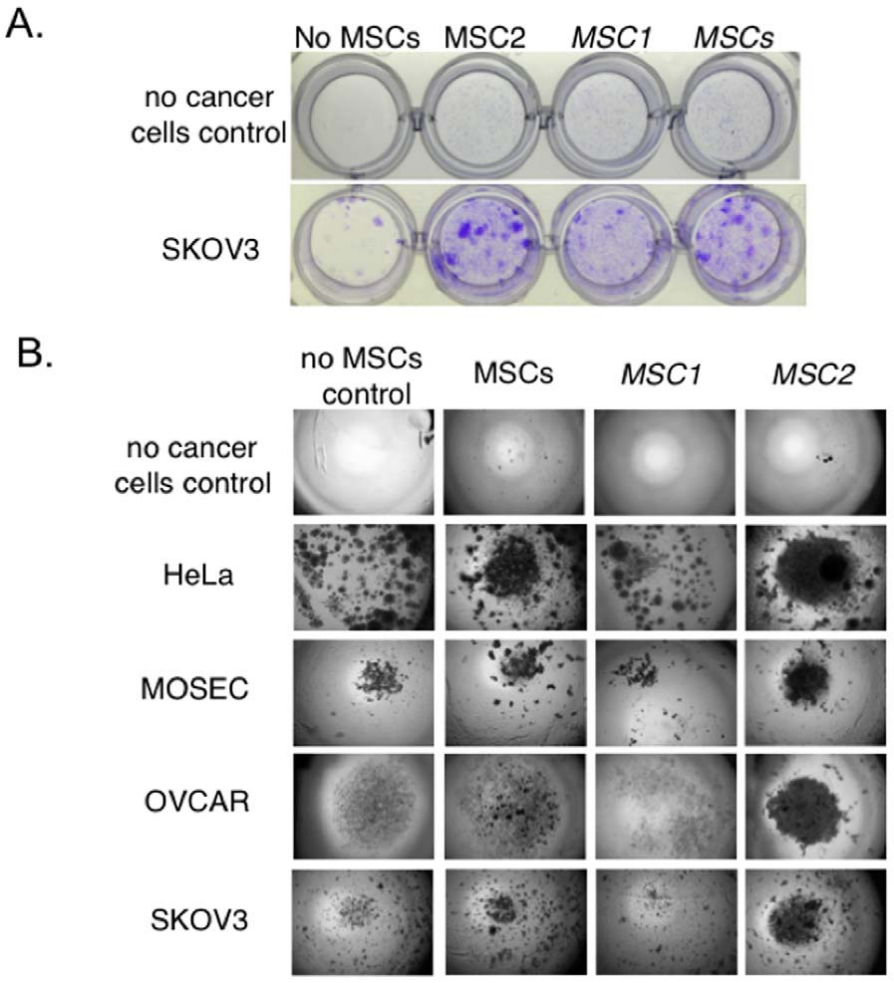
*MSC1* do not support tumor cell growth whereas *MSC2* favor tumor cell growth. **A.** Representative micrographs from colony forming units (CFU) assays performed by culturing human tumor cells (200 cells/well) mixed with MSCs, *MSC1*, or *MSC2* (2 cells/well) at a ratio of 10 cancer cells per 1 MSC and plated in 24-well plates in growth medium supplemented with 10% FBS as indicated in figure. Cultures were grown for 14 days at 37°C in a humidified atmosphere of 5% carbon dioxide balance air. Growth medium was changed every 3–4 days. Colonies were visualized by staining with a crystal violet solution (0.5% crystal violet/10% ethanol). The resulting colonies were enumerated by the colony counting macro in ImageJ software, SKOV3- ovarian adenocarcinoma cell lines. Colony counts are given below the micrographs. Data are representative of at least three independent experiments with at least four MSC donors. **B.** Representative micrograph of tumor spheroids formed by culturing tumor cells (200 cells/well) mixed without any other cells (–) or with MSCs, *MSC1,* or *MSC2* (20 cells/well) at a ratio of 10 cancer cells per 1 MSC and plated over 1.5% agarose in 96-well plates in growth medium supplemented with 10% FBS as indicated in figure. Cultures were grown for 14 days at 37°C in a humidified atmosphere of 5% carbon dioxide balance air. Growth medium was changed every 3–4 days. Micrographs shown represent 20Xmagnified field of the 96-well plate. Cancer cell lines used are: HeLa-human cervical adenocarcinoma, OVCAR-human ovarian adenocarcinoma, SKOV3-human ovarian adenocarcinoma, and MOSEC-murine ovarian surface epithelium carcinoma cells. Data are representative of at least three independent experiments with at least four MSC donors.

**Figure 2:**
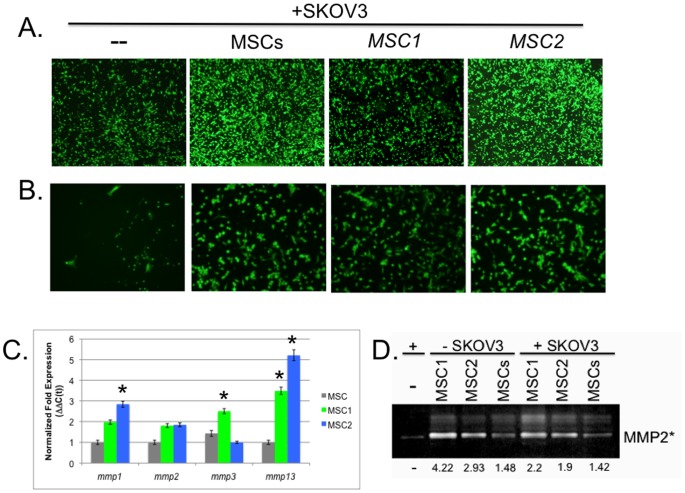
Migration and Invasion of Cancer Cells following MSC phenotype co-culture. Transwell migration and matrigel invasion assays were performed with 3 µM Falcon fluoroblok transwell inserts as described previously [12], [20], [45]. MSCs were added at a 10∶1 ratio of SKOV3 to MSC. These were co-cultured on traditional 2D dishes 72 hr prior to placing the dissociated cells within the transwell inserts. Representative micrographs of **A.** transwell migrating and **B.** matrigel invading cells were visualized and obtained on an inverted fluorescence microscope (A. 100X and B. 200X, Olympus, MetaMorph analysis software). Data are representative of duplicates in at least three independent experiments**. C.** Representative bar graph of quantitative real-time PCR (qPCR) assays carried out as previously described [39]. Gene expression of *mmps* among the MSC samples is expressed by the normalized cumulative threshold method (ΔΔC(t)). *P<0.05 versus the normalized values for MSC. Statistically significant differences were not measured among the other samples. Samples were run in triplicate for at least four different MSC donors. **D.** Representative micrograph following gelatin zymography of the condition medium from MSC-SKOV3 co-cultures (1∶10) or SKOV3 and MSC samples cultured alone as indicated for 72 hr. Bands are of pro-MMP2 (72 kDa) and active MMP2* (62 kDa). The numbers below micrograph are the fold changes relative to SKOV3 alone sample obtained following densitometric analysis (ImageJ). Data are representative of at least three independent experiments.

To further extend our studies on the role of MSCs and ovarian tumors we initially investigated the effect of the recently described *MSC1* and *MSC2* phenotypes on various cancer cell lines [7], [12], [13], [20]. The effect of MSCs, *MSC1,* or *MSC2* on the growth of various cancer cell lines was determined by traditional 2D-colony forming units (CFU) and 3D- tumor spheroid formation assays (Figure 1). Please note that the ratio of cancer cells to MSCs used was 10 to 1 respectively. As expected co-culture with MSCs led to more breast (MDA-MB-231), pancreas (PANC-1) and ovarian (OVCAR, SKOV3, MOSEC) cancer cell colonies and larger tumor spheroids compared to untreated controls (Fig. 2A, B Figures S1 and S2, and data not shown). By contrast, *MSC1*-cancer co-culture consistently led to fewer colonies and much smaller tumor spheroids. Each cancer cell line exhibited their own unique morphology when grown in the CFU and tumor spheroids. It is expected that at a 10∶1 cancer cell to MSC ratio the body of the colonies and spheroids are primarily composed of the cancer cells. This is supported by the observed unique morphologies recorded for each cancer cell line treated with the MSCs. *MSC2* co-culture resulted in the greatest number of CFUs and largest spheroids. We noted that typically the MSCs and *MSC2* co-cultures led to bigger and more diffuse colonies and spheroids whereas the *MSC1* resulted in smaller, tighter, and more compact CFUs and tumor spheroids. CellTracker green labeled MSCs and *MSC2* in the tumor spheroid assays mostly distributed throughout the spheroids (Figure S2). These *in vitro* assays’ results suggest that MSCs and *MSC2* support tumor cell growth whereas *MSC1* seem to diminish tumor cell growth.

We also measured the cytokines, chemokines, and other bioactive factors secreted into the medium by the MSC-cancer cell co-cultures as before (Table 1, [13], [20]). In these experiments we have no means of distinguishing which cell; MSC or cancer, is contributing the bioactive factors, we can simply detect the net effect of the co-culture conditions used here. SKOV3 ovarian cancer cells were plated on 24-well plates until they reached 50–70% confluence. *MSC1*, *MSC2,* (25,000 cells/insert) or medium control were then added into 0.4 µM (no cancer cell-MSC contact) or 8 µM transwell inserts and the co-cultures were allowed another 72 hr prior to collecting the conditioned medium and testing by BioPlex assay. *MSC1*-treated samples elaborated higher levels of pro-inflammatory factors including IL17, IL3, MIG, MIP1β and GM-CSF whereas *MSC2*-treated samples had marked increases in ILRA, IL10, CXCL1, CCL5 and CXCL10 (Table 1). Interestingly, TNF-related apoptosis-inducing ligand (TRAIL) expression was dramatically induced in *MSC1*-treated co-cultures when compared to *MSC2*-treated ones. By contrast, the expression of GM-CSF, LIF, and TRAIL was attenuated in *MSC2*-treated samples when compared to *MSC1*-treated ones. We observed similar trends when we sampled the biofactor secretion from the 3D tumor spheroid co-cultures (data not shown).

**Table 1:** Ovarian cancer cells co-cultured with *MSC1* differ from *MSC2* co-cultures in their secretion of bioactive factors.

Bioactive Factor	Contactdependenteffect	*MSC1*	*MSC2*
IL1RA	**–**	↓	↑↑
IL3	**–**	↑↑	↓
IL10	**+**	**–**	↑
IL12p40	**–**	↓	↑
IL17	**+**	↑↑	↑
CXCL1 (Groα)	**–**	↓	↑↑
CXCL10 (IP10)	**+**	↓	↑↑
CCL5 (RANTES)	**+**	−/↓	↑↑
MIG	**+**	↑	**–**
MIP1β	**+**	↑	↑↑
GM-CSF	**+**	↑↑	↓
HGF	**+**	↓	**–**
LIF	**–**	↑↑	↓
TRAIL	**+**	↑↑	↓

SKOV3 ovarian cancer cells were plated on 24-well plates until they reached 50–70% confluence. *MSC1*, *MSC2,* (25,000 cells/insert) or medium control were then added into 0.4 µM (no cell-cell contact) or 8 µM transwell inserts and the co-cultures were allowed another 72 hr prior to collecting the conditioned medium and testing by Bio-Plex Cytokine Assays following the manufacturer’s instructions (Human Group I & II; Bio-Rad, Hercules, CA). Arrows represent relative normalized changes compared with the SKOV3 alone control. Biofactor levels that were different between the MSCs grown in 0.4 µM (no cell-cell contact) versus 8 µM transwell inserts are represented by “+.” Those biofactor levels that were similar in both sample groups are represented by “−.” Data are representative of triplicate measurements with 4 MSC donors in at least 4 independent experiments.

### Migration and Invasion of Cancer Cells Following MSC Phenotype Co-culture

We next examined the effect on the migration and invasion capabilities of these cancer cells following co-culture with the MSCs, *MSC1,* and *MSC2*. Similar to the previous report that conventionally derived MSCs promote MDA-MB-231 breast cancer cell migration and invasion [8], we also found that migration and invasion was promoted by MSCs and *MSC2* but not by *MSC1* (Figure 2). We observed about a two-fold increase in both migration and invasion assays by MSCs and *MSC2* co-culture (Figure 2A and B, respectively). In our experiments, all MSCs were added at a 10∶1 ratio of cancer cells to MSC as before. We tested the effect of co-culture of the cells plated in traditional 2D dishes 72 hr prior to placing the dissociated cells within the transwell inserts. We also tested the effect of the MSCs on the 3D tumor spheroids grown cancer cells after subsequent dissociation and loading in transwell inserts for these assays (Figure 2A and B). We recorded similar effects by the MSCs on the invasion and migration of the cancer cells regardless of culturing conditions.

Additionally, the effect of the MSCs, *MSC1,* and *MSC2* in these assays does not appear to correlate with their expression of matrix metalloproteinases (MMPs, Figure 2C and D). We consistently measured increased expression of several *mmp*s following MSCs induction into the *MSC1* or *MSC2* phenotypes (Figure 2C). We also observed elevated secretion of activated MMP2 (MMP2*) into the condition medium of co-cultures of *MSC1* and *MSC2* with SKOV3 when compared with medium from cultures with MSCs or SKOV3 alone (Figure 2D). Although, these levels were slightly lower than those of the MSCs samples without cancer cell co-cultures (Figure 2D). These results indicate that the distinct MSC-mediated effects on cancer migration and invasion are more complex and perhaps not directly mediated by MMP2* in agreement with the studies of the report described earlier [8].

### *MSC1* Attenuate Tumor Growth Whereas *MSC2* Promote Tumor Growth and Metastasis

**Figure 3:**
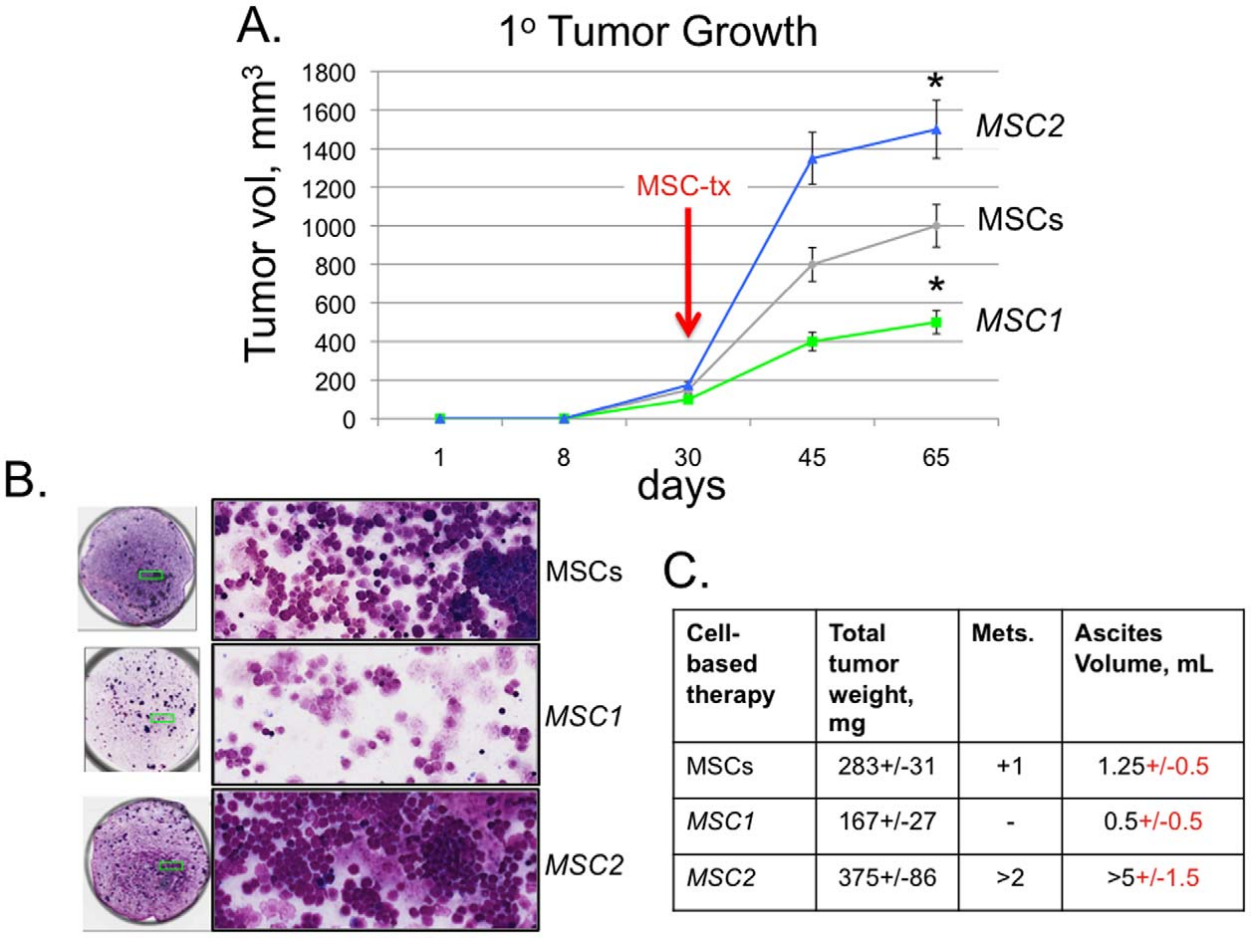
*MSC1* do not support tumor growth whereas *MSC2* favor tumor growth and metastasis. The established syngeneic mouse model for epithelial ovarian cancer used is based upon a spontaneously transformed mouse ovarian surface epithelial cell (MOSEC) line ID8 that has been previously described [21]. At approximately 4 weeks a single dose of human MSCs (MSCs), *MSC1*, or *MSC2* (1×10^6^/per mouse) were injected intraperitonealy (IP) as indicated by red arrow. **A.** Tumor growth was measured at weekly intervals until day of mouse sacrifice (Day 65). Harvested tumors and metastasis were weighed, counted and processed for flow cytometry and immunohistochemical analysis (IHC). *P<0.05 versus the MSCs-treated tumors. **B.** Accumulated ascites was collected, measured, and a sample was spun on cytospin slides and stained by DiffQuick cytology stain by standard methods. Left circles are representative micrographs of cytospin slides (20X) with enlarged areas to the right marked by green box (100X). **C.** Table of average +/−SEM results among the different MSC-treatment groups. Data are representative of three independent experiments with at least 6 mice per treatment group.

The anti-tumor *MSC1* and the pro-tumor *MSC2 in vitro* effects were further supported in pilot studies with human ovarian cancer xenograft animal models treated with the MSC-based therapies as previously established ([7] and data not shown). We subsequently used the immune *competent* MOSEC model to verify these MSC-tumor effects (Figure 3, [21]). The tumors were established in the mice with 1×10^7^ MOSEC (ID8) cells. After approximately 4 weeks a single dose of CellTracker fluorescently labeled human MSCs, *MSC1*, or *MSC2* (1×10^6^/per mouse) were injected IP. The small amount of remaining MSCs preparations within the syringes were again plated and observed for contamination and subsequent growth properties. No change was noted among these spent MSC preparations in growth properties even after 2-weeks of culture.

Following 24 hr after the MSC-based treatments, one animal was sacrificed per treatment group to measure MSC engraftment to the primary tumor. All MSC-treated samples had similar detectable pre-labeled fluorescence MSCs within the tumor tissue trending towards more *MSC1* and *MSC2* measured than MSCs with approximately 15–25 cells versus 10–15 counted per 200X field after 24 hr of MSC-treatment (data not shown). Based on the literature and our previous experiments, MSC-based therapy of the tumor typically results in very little engraftment (<0.5%) or local proliferation of MSCs at the tumor site [10], [14]. Tumor growth was measured at weekly intervals until day of mouse sacrifice (Day 65). At harvest, the ascites accumulated in the tumor bearing mice was collected. The tumors and metastases were measured and processed for flow cytometry and IHC analyses [7].

The collected ascites samples were spun down on cytospin slides and stained with Diff Quick (Figure 3B). Notably, very little (<0.5 mL/mice) to no ascites accumulated in *MSC1*-treated animals compared with MSCs- (1.25 mL/mice) and *MSC2*-treated (>5 mL/mice) animals (Figure 3C). Furthermore, *MSC2*-treated animals had the most tumor cell aggregates within the ascites followed by the MSC-treated samples, with few tumor aggregates found in *MSC1*-treated sample ascites (Figure 3B). In parallel, the tumor size and weights were biggest in *MSC2*-treated (∼1500 mm^3^ and 375 mg) animals followed by MSCs-treated animals (∼1000 mm^3^ and 283 mg) and *MSC1*-treated animals (∼500 mm^3^ and 167 mg, Figure 3A and C). Metastasis was found only in MSCs- and *MSC2*-treated mice.

### Tumor-associated Leukocytes Differ among the MSC-treated Groups

**Figure 4:**
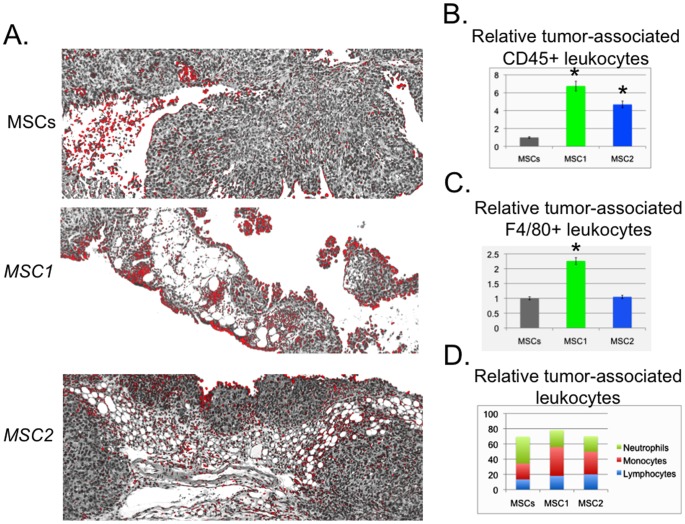
Tumor-associated leukocytes differ among the MSC-treated groups. MOSEC tumors were established in C57BL/6 mice for 4 weeks. MSCs, *MSC1,* or *MSC2* (1×10^6^ in 0.5 mL HBSS) were infused IP and the mice were harvested after 65 days. Tumors were excised, fixed, and cut into 5 µM sections and processed for antibody staining by standard methods or single cell suspensions were made from the tumors and processed for flow cytometry analysis [7]. Data are representative of three independent experiments with at least 6 mice per treatment group. **A.** Representative micrographs of the tumor sections processed by IHC, stained with DAB, and then recorded with the Aperio ScanScope (40X, Aperio, Vista, CA). Shown is the subsequent ImageJ threshold analysis with CD45+cells colorized red. **B.** Bar graph depicting the results from the CD45+ flow cytometry analyses of the tumors relative to the MSC-treated tumors. *P<0.05 versus the MSCs-treated tumors. Statistically significant differences were not measured between *MSC1-* and *MSC2*-treated tumor samples. **C.** Bar graph depicting the results from the F4/80+ flow cytometry analyses of the tumors relative to the MSC-treated tumors. *P<0.05 versus the MSCs-treated tumors. Statistically significant differences were not measured between MSCs*-* and *MSC2*-treated tumor samples. **D.** Bar graph depicting the results from flow cytometry analyses to identify neutrophil, monocyte, and lymphocyte populations among the tumor samples as described in Materials and Methods. Flow cytometry data are representative of at least duplicate samples from at least three independent experiments.

Flow cytometry and IHC analyses of harvested tumors demonstrated some interesting differences dependent upon the MSC-treatments (Figure 4). Based on both CD45+ flow cytometry and IHC analyses *MSC1*-treated groups appeared to have the greatest recruitment of leukocytes to the TME compared to the other treatment groups (Figure 4A and B). *MSC2*-treated groups also had an increased number of tumor-associated CD45+leukocytes compared to MSC-treated groups. Representative micrographs of the ImageJ threshold analysis with CD45+cells colorized red demonstrate these differences (Figure 4A). Additionally, *MSC1*-treated groups had elevated levels of F4/80+ leukocytes (likely macrophages) compared to MSCs- and *MSC2*-treated groups as determined by flow cytometry (Figure 4C). The MSCs-treated groups had the most tumor-associated neutrophils (∼35%) whereas *MSC1*-treated groups had more monocytes (∼40%) and *MSC2*-treated groups had close to equivalent numbers of neutrophils, monocytes and lymphocytes (∼20%/each) based on differential flow cytometry analyses with specific antibodies to CD3, CD4, CD8, CD11b, CD45R, Ly-6G (Gr-1), and NKG2D (CD314) (http://phenome.jax.org/db/q?rtn=projects/docstatic&doc=Jaxpheno6/Jaxpheno6_Protocol).

**Figure 5:**
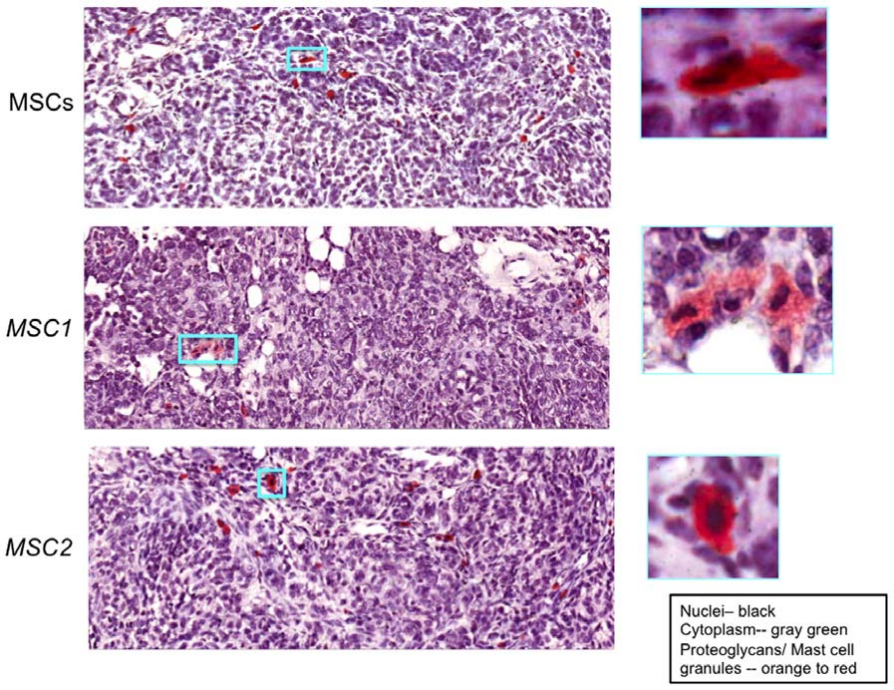
Proteoglycan-rich stained mast cells found in tumor sections from *MSC2*- and MSC-treated tumor groups but mostly degranulated ones found in *MSC1*-treated tumor groups. MOSEC tumors were established in C57BL/6 mice for 4 weeks. MSCs, *MSC1,* or *MSC2* (1×10^6^ in 0.5 mL HBSS) were infused IP and the mice were harvested after 65 days. Tumors were excised, fixed, and cut into 5 µM sections by standard methods [7]. Sections were processed for safranin O proteoglycan staining (www.ihcworld.com). Representative micrographs of several MSC-treated tumor sections are included from images obtained from the Aperio ScanScope (200X, Aperio, Vista, CA). The expected color for each tissue element is described in the inset on the lower right hand side. 400X images are included in boxed insets. Data are representative of three independent experiments with at least 6 mice per treatment group.

Next, we used a proteoglycan-specific stain (safranin O-fast green) to help visualize the mast cells (MCs) found within the MSC-treated tumor sections (Figure 5). MCs are immune cells that are increasingly implicated in tumor growth, spread, and aggressiveness [22]. The metastatic potential of tumors is affected by the composition of the tumor associated extracellular matrix (ECM). MCs are known to promote ECM protein deposition and are associated with various human ECM disorders [23], [24]. Lastly, MCs are also known to interact with MSCs [3], [25]. Although we did not observe obvious differences in the number of safranin O positive mast cells in each of the MSC-treated groups, there appeared to be differences in the stained granules within the MCs among them. Specifically, while MSC- and *MSC2*-treated tumor sections appeared to contain mostly safranin O-positive granule laden MCs, *MSC1*-treated tumor sections contained mostly MCs that appeared degranulated (insets of Figure 5). We also noted that the MCs were distributed mostly throughout the stromal fibrovascular compartments of all tumors where they may also be acting to affect the ECM (Figures S3 and S4). These results indicate that the anti-tumor *MSC1*-effects and the pro-tumor *MSC2*-effects may be mediated by differences in their ability to distinctly affect various tumor-associated leukocytes as well as directly or indirectly affect the ECM content of the tumor microenvironment.

## Discussion

The novel finding of this study is that mesenchymal stem cells (multipotent stromal cells, MSCs) induced into the *MSC1* phenotype attenuate cancer cell growth while MSCs induced into the *MSC2* phenotype mostly mimic conventional MSCs in promoting cancer cell growth and spread. Additionally, that once the *MSC1* and *MSC2* phenotypes are induced and reintroduced they appear to lead to distinct tumor effects. In another complementary study, we similarly tested for the stability of the induced phenotypes and their distinct therapeutic effects in a murine model of pain [14].

Recently, a shadow has been cast over the successful and increasing use of MSC-based therapies in many diseases, by the growing controversy of whether the MSCs used in the treatment might promote tumor growth as some preclinical studies, including ours, suggest [7]. By contrast, others have argued that MSCs attenuate tumor growth and spread. However, most agree that as a result of the propensity of MSCs to home to tumors, these cells used in cell therapies of cancer provide ideal cancer drug delivery vehicles [4]–[6], [26]. In this study, we present evidence that might shed some light over these controversies and that may provide some guidance in the design of safer MSC-based therapies.

We extended our work on MSCs and ovarian cancer, as well as our study describing a new approach for the induction of MSCs into a pro-inflammatory *MSC1* and an immunosuppressive *MSC2* phenotype. Accordingly, we chose to focus our investigation on the distinct effect that *MSC1* and *MSC2* might have on tumor growth and spread compared to the established one with conventionally prepared MSCs [7], [13]. Our initial *in vitro* experiments demonstrated that *MSC1* co-culture with various cancer cells diminished their capacity to form colonies in contrast to growth promoting MSC- or *MSC2*-co-cultures (Figure 1 and Figures S1 and S2). This effect remained constant even when tested by 3D tumor spheroid models. In this study we only tested cancer cells derived from solid organ tumors and not from leukemia or other blood-related malignancies. We also used MSC to cancer cell ratios of 1∶10 throughout the study to more closely resemble the proportions that might be achieved in the clinic with MSC-based therapies and different to the 1∶1 ratios used by other MSC and cancer studies (e.g. [8], [27]–[29]).

MSCs targeted to cancers are expected to contribute many bioactive factors once resident in the TME, such as mitogens, extracellular matrix (ECM) proteins, angiogenic, and inflammatory factors, as well as exosomes or microvescicles. MSCs are also expected to affect tumor-associated leukocytes either directly by cell-cell contact or indirectly by these secreted factors [3]. Most of these parameters were measured in this study. We previously reported that there were differences among several of these secreted bioactive factors following the induction of MSCs into *MSC1* and *MSC2* [13]. Co-cultures of these MSC phenotypes with the cancer cells also reflected distinct effects on the secreted factors as summarized on Table 1. Both contact-dependent and independent effects were observed. Increases measured in the levels of CCL5 (RANTES) secreted by the pro-tumor *MSC2* groups are in agreement with previous reports [8], [13]. By contrast, *MSC1* treatment groups had elevated levels of IL17, GM-CSF, and TRAIL that would suggest an overall inflammatory and pro-apoptotic effect by these cells. *MSC2* treatment groups also had elevated levels of secreted IL1RA, IL10 and most chemokines tested, which suggests a net tumor supportive immunosuppressive effect by this treatment group [27]. However, it is important to recognize that the expression of bioactive factors is by necessity a dynamic process, quickly changing at any given time and place and probably confined to communication across short intercellular distances. We are also not able to distinguish the source be it MSC or cancer cell of the factors elaborated in our established co-culture experimental conditions. Furthermore, what we are able to measure with the current technology is one snapshot of time and thus it must be accordingly weighed and validated with other supportive experiments prior to drawing too many conclusions.

To this end, transwell migration and matrigel invasion capabilities were also studied (Figure 2). However, though we measured fewer migrating and invading cells for the *MSC1* sample groups compared to the other MSC sample groups, we could not attribute this difference to decreased expression of activated MMP2. Additionally, we have not been able to detect significant levels of either the zymogen or active forms of MMP9 in MSC phenotype *in vitro* cultures or co-cultures with cancer cells. These results are intriguing given the documented importance of MMP2 and 9 in tumor spread and invasion [30]. Further studies are needed to investigate this complex tumor process and how the MSCs might affect it.

Following these *in vitro* experiments, we next investigated the effects of the MSC-based therapies in an immune competent mouse model of ovarian cancer that has been useful in similar studies [21], [31], [32]. Since the most prevalent effect of MSC-based therapy reported in human clinical trials appears to be immune modulation, and the profile of bioactive factors primarily expressed by MSCs are immune modulatory, we thought it important to use immune *competent* models [2], [33]. Previous studies with human MSCs introduced into allogeneic or xenogeneic hosts have been similarly reported with success [1], [9], [34]. In this context, we consistently observed that the *MSC1*-treatment groups had smaller tumors without any detectable metastasis, and accumulated little to no ascites when compared to the MSCs- or *MSC2*-treated groups (Figure 3). Upon staining of the collected ascites, it was evident that there were large tumor aggregates or spheroids present in the MSCs- and *MSC2*-treatment groups but not in the *MSC1* ones. MSC-based therapies of tumors or other diseased organs typically results in very low engraftment by the delivered MSCs. It is established that one hurdle in the translation of MSC-based therapies remains improving their survival in the recipient host [1], [9], [34].

We used both flow cytometry and immunohistochemical analyses to determine the changes among the treatment groups in the tumor-associated leukocytes (Figures 4 and 5). Here, too, we found changes among the MSC-treated groups as was expected. The CD45+population of cells present in the tumors were more numerous in *MSC1*- and *MSC2*- treatment groups than in MSCs-treated groups. Additionally we measured the greatest number of F4/80+cells in the *MSC1*- treated group compared to the others. The significance of these findings remains to be elucidated. Tumor-associated macrophages (TAMs) are known to be educated from tumor eradicating cells to tumor promoting cells with F4/80 expression potentially changing from one population to the other [35]–[37]. It will be interesting to determine in future studies whether tumor-associated MSCs and TAMs directly affect each other and can be “re-educated” from one form to the other following this interaction.

Macrophages, mast cells (MCs), and MSCs also affect ECM proteins, yet another component of the TME important to tumor growth and spread [23], [24], [30], [38]. Thus, the changes in mast cells and collagen (ECM) levels among the MSC-treated tumor groups were measured (Figure 5 and Figure S3). Safranin O stains the proteoglycan-rich granules of mast cells and surprisingly revealed that the MCs of tumor sections of MSCs- and *MSC2*-treated groups were mostly loaded with these granules while the *MSC1*-treated groups were not. Furthermore, we observed localization of the MCs to the stromal compartments of the tumors, which may suggest an association of MCs and the ECM. This association was further implicated by comparison of the safranin O stained sections with those of the Verhoeff-Van Gieson (VVG) collagen stained sections, which revealed mast cells concentrated in areas with the darkest pink/red collagen stained regions (Figure S4). Unexpectedly, we observed the opposite effect of *MSC1* on collagen levels (*in vivo*) than we previously reported for *MSC1* induction alone (*in vitro*) [13]. VVG stained tumor sections from *MSC1*-treated groups had less dark pink/red areas than the other samples, whereas *in vitro MSC1* had the greatest expression of collagen compared to the other samples. These differences may be explained by direct *in vivo* interactions between the MSCs and MCs that were recently discovered and that would be present in the TME but lacking in the *in vitro* setting [25]. Further investigation of the interaction of MCs with MSCs within the TME will have to be added to those of MSCs and macrophages mentioned above. Adding to the complexity of the TME, MSCs, macrophages, and MCs seem to share many properties affecting the secretion of bioactive factors and the tumor immunity [26], [39]–[43].

More detailed analyses are required to complete our understanding of the effect that MSC-based therapies might have on all of the tumor-associated leukocytes including MCs and macrophages. In particular, it would be interesting to begin to dissect the contributions of each leukocyte population in the MSC-affected tumors by using specific mouse knockout models. We also expect that the study of other solid tumor and leukemia models as well as other strains of mice may identify subtle differences in the net effect of the MSC-based therapies that will be useful to our understanding of the TME and its contribution to tumor growth and spread. Important as well will be determining the most effective MSC-based cancer therapy. To this end, the optimal dose, frequency, and timing of the MSC-based therapy need to be determined for each cancer. We are encouraged that the *ex vivo* induced *MSC1* and *MSC2* phenotypes appear to remain stable when re-introduced into various animal disease models and were capable of mediating distinct results even 65 days after just a single MSC injection (Figure 3).

Cell-based therapies are undoubtedly gaining ground given their growing international use, regulatory agency approval (FDA and European Medicines Agency-EMA), billion dollar a year market, and proven efficacy in many human diseases [44]. Among these, *MSC*-based therapies are widely used because MSCs are thus far clinically safe, are easily obtained from adult tissues, can be expanded as well as stored, and are unique in their immune modulating capabilities. Additionally, their proclivity for the tumor microenvironment makes them ideally suited for the directed delivery of anti-cancer payloads. An ideal therapeutic approach for the complex pathology of cancer may be a complementary one that employs conventional methods to target the cancer cells (seed) combined with MSC-based therapies that target the TME (soil). Finally, the new *MSC1*- and *MSC2*-therapy approach we have identified provides a convenient tool with which to begin to dissect the contribution of MSCs to tumors, and may help resolve some of the surrounding controversies to safely advance the use of MSC-based therapies in many human diseases including cancer.

## Materials and Methods

### Cells

Bone marrow-derived human MSC (MSCs) used for all studies were obtained from the Tulane Center for Stem Cell Research and Regenerative Medicine, New Orleans, LA or Lonza, Walkersville, MD and are IRB exempt as previously described [13]. MSCs from at least six different human donors were used in these experiments and cultured as previously described [12], [45]. All experiments were conducted on MSCs at a passage ≤5. HeLa human cervical adenocarcinoma, OVCAR-human ovarian adenocarcinoma, and SKOV3 (SKOV3AB) human ovarian adenocarcinoma were obtained from the American Type Tissue Collection (ATCC, Walkersville, MD). PANC-1 human pancreatic adenocarcinoma, and SKOV3FM were obtained as a gift from Dr. Frank C. Marini (Wake Forest Medical Center, NC and are also commercially available from ATCC, Walkersville, MD). Preparation of MSCs into a pro-inflammatory *MSC1* phenotype or an immunosuppressive *MSC2* phenotype was described previously ([13], patent-pending US 61/391,749).

### Animals

Animal care and use was pre-approved by the Tulane University Medical Center Advisory Committee for Animal Resources. 3–7-week-old female C57BL/6J *wt* mice were obtained from Jackson Laboratories (Bar Harbor, ME). The syngeneic mouse model for epithelial ovarian cancer based upon a spontaneously transformed mouse ovarian surface epithelial cell (MOSEC) line ID8 has been previously described [21], [46]. ID8 cells were a generous gift from Dr. Katherine F. Roby (Kansas University Medical Center). At approximately 4 weeks post cancer cell introduction and tumor formation, 1×10^6^ cells/per mouse of CellTracker fluorescently-labeled wt MSCs, *MSC1*, *MSC2* or mock control was infused IP (Molecular Probes, Life Technologies, Carlsbad, CA) [47]. The ability of the cells to reach their target was measured by flow cytometry analyses of collected tumors 24 hr after the MSC infusions [47]. The 24 hr window was chosen as optimal for MSC engraftment measurements based on previous studies [47]. Mice were monitored daily for changes in weight, morbidity, and mortality. Tumors were measured and at harvest, ascites, tumors and any metastases were weighed and documented as before [7]. Kaplan-Meier survival plots of mice were analyzed by the log rank test (Prism4, GraphPad Software Inc. CA). Greater than 6 mice per sample group was used in each of the experiments.

### Flow Cytometry

Single cell suspensions of collected tumors were achieved by the method previously described [32]. Analysis of cell surface markers expressed from the obtained tumor samples was done by multi-color cell surface antibody staining as in that study, or as indicated for the specific cell subsets including anti-CD3, -CD4, -CD8, -CD11b, -CD11c, -CD19, -CD45R, - Ly-6G (Gr-1), and -NKG2D (CD314) [46], [48]. Intracellular cytokine antibody staining was achieved after fixation and permeabilization of the cells. Isotype controls and untreated or unstained samples were routinely run in parallel as standard. End point flow cytometry analysis was performed on a BD LSRII analyzer and analyzed with CellQuest software. Data are representative of at least duplicate samples from at least three independent experiments.

### Colony Forming Units (CFU) and Tumor Spheroid Assays

CFU assay was performed by culturing human tumor cells (200 cells/well) mixed with conventionally prepared MSCs, *MSC1* or *MSC2* (2 cells/well) at a ratio of 10 cancer cells per MSC and plated in 24-well plates in growth medium supplemented with 10% FBS as indicated. Cultures were grown for 14 days at 37°C in a humidified incubator. Growth medium was changed every 3–4 days. Colonies were visualized by staining with a crystal violet solution (0.5% crystal violet/10% ethanol). The resulting colonies were enumerated by the colony counting macro in ImageJ software. Tumor spheroids were formed by culturing tumor cells (200 cells/well) mixed without any other cells (–) or with CellTracker labeled MSCs, *MSC1* or *MSC2* (20 cells/well) at a ratio of 10 cancer cells per 1 MSC and plated over 1.5% agarose in 96-well plates in growth medium supplemented with 10% FBS as indicated. Cultures were grown for 14 days at 37°C in a humidified incubator. Growth medium was changed every 3–4 days. Micrographs shown represent a 20-fold magnified field of the 96-well plate. CFU and tumor spheroid assays were performed in at least three independent experiments with duplicate wells.

### Migration and Invasion Assays

Migration and invasion assays were performed with cells loaded on 3 µM Falcon fluoroblok transwell inserts and allowed 16 hrs in a humified CO2 incubator as described previously [12], [20], [45]. Transwell migrating and matrigel invading cells were visualized on an inverted fluorescence microscope (Olympus). Image analyses were routinely performed with ImageJ. Data are representative of duplicates in at least three independent experiments.

### qPCR

Quantitative Real-Time PCR (qPCR) was carried out as previously described using the following primers pairs [39]: matrix metalloproteinase 1 *(mmp)1-*forward (F) GGA GAT CAT CGG GAC AAC TC *; mmp1-*reverse (R*)-*ACC GGA CTT CAT ATG TCG *; mmp2*-F-CAA GTG GTC CGT GTG AAG TAT G; *mmp2*-R-CGT CAT CGT AGT TGG CTG TG; *mmp3*-F-GAC AAA GGA TAC AAC AGG GAC C; *mmp3*-R-TAT CAG AAA TGG CTG CAT CG; *mmp9*-F-CAA GGA TGG GAA GTA CTG GCG; *mmp9*-R- TCA ACT CAC TCC GGG AAC TC; *mmp13*-F- GAT ACG TTC TTA CAG AAG; *mmp13*-R GAC AAA TCA TCT TCA TCA CC; membrane-type matrix metalloproteinase-1 (*mt-mmp)1*-F-GTC TTC AAG GAG CGC TGG TTC TG *mt-mmp1*-R- TAG CCC GGT TCT ACC TTCA G; *18S rRNA –*F-GAG GGA GCC TGA GAA ACG G, *18S rRNA* -R-GTC GGG AGT GGG TAA TTT GC-3′ (IDT, Coralville, IA). Samples from at least three independent experiments were run in triplicate.

### Histology and Immunohistochemistry

The collected ascites samples were spun down on cytospin slides and processed for Diff Quick stain as described (http://www.ihcworld.com/_protocols/special_stains/diff_quick_ellis.htm). Tumors were fixed in 10% formalin solution and embedded in paraffin by standard methods. Sections were cut into 5 µm sections and stained with hematoxylin and eosin (H&E), Verhoeff-Van Gieson (VVG)– elastic fiber/collagen staining and safranin O– proteoglycan staining were performed also as described (www.ihcworld.com). Immunostaining was performed using monoclonal anti-hCAP-18/LL-37, -CD45, -F4/80, and other relevant markers as before [49]. All stained tissue sections were scanned with the Aperio ScanScope (Aperio, Vista, CA) at an initial magnification of 40X, and images were visualized and captured using the Aperio ImageScope program. Image analyses were routinely performed with ImageJ. For threshold analysis (percent DAB or safranin positive), the images were digitally adjusted to remove background and increase the contrast between the tissue and the background. The RGB images were stacked into separate R, G, B images and threshold determinations were used to digitally highlight all the stained tissue while dismissing the background. Finally, the percent of highlighted pixels (positive cells) was calculated relative to total area of the field. A similar ImageJ analysis method was used to determine collagen positive areas within the VVG stained tumor sections as detailed in http://cardprint.ucsd.edu/CV_Lab_Web_Page/HowToDocs/ImageJProtocol.pdf. Greater than 10 viewing fields were recorded and analyzed after three independent experiments for each sample group.

### Statistical Analysis

Data are presented as average +/− standard error of the mean (S.E.M.). Multiple group comparison was performed by one-way analysis of variance (ANOVA) followed by the Bonferroni procedure for comparison of means. Comparison between any two groups was analyzed by the two-tailed Student's t-test or two-way ANOVA (Prism4, GraphPad Software Inc. CA). Values of *P*<0.05 were considered statistically significant.

## Supporting Information

Figure S1 **MSC1 diminish tumor growth whereas MSC2 favor tumor growth.** Tumor spheroids were formed by culturing tumor cells (200 cells/well) mixed without any other cells (–) or with CellTracker green labeled MSCs, *MSC1,* or *MSC2* (20 cells/well) at a ratio of 10 cancer cells per 1 MSC and plated over 1.5% agarose in 96-well plates in growth medium supplemented with 10% FBS as indicated in figure. Cultures were grown for 14 days at 37°C in a humidified atmosphere of 5% carbon dioxide balance air. Growth medium was changed every 3–4 days. Representative micrographs shown represent 20X magnified bright field of the 96-well plate. Cancer cell lines used are: HeLa- human cervical adenocarcinoma, PANC-1- human pancreatic adenocarcinoma, OVCAR-human ovarian adenocarcinoma, SKOV3-human ovarian adenocarcinoma, and MOSEC-murine ovarian surface epithelium carcinoma cells.
(TIF)

Figure S2 **MSC1 diminish tumor growth whereas MSC2 favor tumor growth.** Fluorescence micrographs corresponding to those bright field micrographs presented in Figure S1. CellTracker green labeled MSCs, *MSC1,* or *MSC2* appear as the brighter spots in the images. It appears that the cells distribute throughout the tumor spheroids–whose shadows are visible in these fluorescence micrographs.
(TIF)

Figure S3 ***MSC1*** **-treated tumor samples have diminished levels of collagen within the TME compared to** ***MSC2*** **- and MSC-treated tumor groups.** MOSEC tumors were established in C57BL/6 mice for 4 weeks. MSCs, *MSC1,* or *MSC2* (1×10^6^ in 0.5 mL HBSS) were infused IP and the mice were harvested after 65 days. Tumors were excised, fixed, and cut into 5 µM sections by standard methods [7]. Sections were processed for Verhoeff-Van Gieson (VVG) elastic fiber/collagen staining (www.ihcworld.com). Representative micrographs of several MSC-treated tumor sections are included from images obtained from the Aperio ScanScope (40X, Aperio, Vista, CA). The expected color for each tissue element is described in the inset on the lower right hand side. 80X images are included in boxed insets. Data are representative of three independent experiments with at least 6 mice per treatment group.
(TIF)

Figure S4 **Co-localization of tumor associated mast cells with collagen.** MOSEC tumors were established in C57BL/6 mice for 4 weeks. MSCs, *MSC1,* or *MSC2* (1×10^6^ in 0.5 mL HBSS) were infused IP and the mice were harvested after 65 days. Tumors were excised, fixed, and cut into 5 µM sections by standard methods [7]. Sections were processed for Verhoeff-Van Gieson (VVG) elastic fiber/collagen staining (left panels) or for safranin O proteoglycan staining (right panels, www.ihcworld.com). Representative micrographs of several MSC-treated tumor sections are included from images obtained from the Aperio ScanScope (40X, Aperio, Vista, CA). Yellow arrows indicate comparable sections among the tumor tissue sections. Data are representative of three independent experiments with at least 6 mice per treatment group.
(TIF)

## References

[1] Salem HK, Thiemermann C (2010) Mesenchymal stromal cells: current understanding and clinical status. Stem Cells 28: 585–596. 19967788 10.1002/stem.269 PMC2962904

[2] Tolar J, Le Blanc K, Keating A, Blazar BR (2010) Concise review: hitting the right spot with mesenchymal stromal cells. Stem Cells 28: 1446–1455. 20597105 10.1002/stem.459 PMC3638893

[3] Waterman RS, Betancourt AM (2012) The role of mesenchymal stem cells in the tumor microenvironment: InTech. http://www.intechopen.com/books/tumor-microenvironment-and-myelomonocytic-cells/the-role-of-mesenchymal-stem-cells-in-the-tumor-microenvironment.

[4] Klopp AH, Gupta A, Spaeth E, Andreeff M, Marini F 3rd (2010) Dissecting a Discrepancy in the Literature: Do Mesenchymal Stem Cells Support or Suppress Tumor Growth? Stem Cells. 10.1002/stem.559 PMC3059412 21280155

[5] Klopp AH, Gupta A, Spaeth E, Andreeff M, Marini F 3rd (2011) Concise review: Dissecting a discrepancy in the literature: do mesenchymal stem cells support or suppress tumor growth? Stem Cells 29: 11–19. 21280155 10.1002/stem.559 PMC3059412

[6] Kidd S, Spaeth E, Klopp A, Andreeff M, Hall B, et al (2008) The (in) auspicious role of mesenchymal stromal cells in cancer: be it friend or foe. Cytotherapy 10: 657–667. 18985472 10.1080/14653240802486517

[7] Coffelt SB, Marini FC, Watson K, Zwezdaryk KJ, Dembinski JL, et al (2009) The pro-inflammatory peptide LL-37 promotes ovarian tumor progression through recruitment of multipotent mesenchymal stromal cells. Proc Natl Acad Sci U S A 106: 3806–3811. 19234121 10.1073/pnas.0900244106 PMC2656161

[8] Karnoub AE, Dash AB, Vo AP, Sullivan A, Brooks MW, et al (2007) Mesenchymal stem cells within tumour stroma promote breast cancer metastasis. Nature 449: 557–563. 17914389 10.1038/nature06188

[9] Rayment EA, Williams DJ (2010) Concise review: mind the gap: challenges in characterizing and quantifying cell- and tissue-based therapies for clinical translation. Stem Cells 28: 996–1004. 20333747 10.1002/stem.416 PMC2962908

[10] von Bahr L, Batsis I, Moll G, Hagg M, Szakos A, et al.. (2012) Analysis of Tissues Following Mesenchymal Stromal Cell Therapy in Humans Indicate Limited Long-Term Engraftment and No Ectopic Tissue Formation. Stem Cells. 10.1002/stem.1118 22553154

[11] Coffelt SB, Scandurro AB (2008) Tumors sound the alarmin(s). Cancer Res 68: 6482–6485. 18701469 10.1158/0008-5472.CAN-08-0044 PMC2755533

[12] Zwezdaryk KJ, Coffelt SB, Figueroa YG, Liu J, Phinney DG, et al (2007) Erythropoietin, a hypoxia-regulated factor, elicits a pro-angiogenic program in human mesenchymal stem cells. Exp Hematol 35: 640–652. 17379074 10.1016/j.exphem.2007.01.044

[13] Waterman RS, Tomchuck SL, Henkle SL, Betancourt AM (2010) A new mesenchymal stem cell (MSC) paradigm: polarization into a pro-inflammatory MSC1 or an Immunosuppressive MSC2 phenotype. PLoS One 5: e10088. 20436665 10.1371/journal.pone.0010088 PMC2859930

[14] Waterman RS, Jenny M, Bobby DN, Anna ES, Aline MB (2012) Anti-Inflammatory Mesenchymal Stem Cells (MSC2) Attenuate Symptoms of Painful Diabetic Peripheral Neuropathy. Stem Cells Translational Medicine: 557–565. 10.5966/sctm.2012-0025 PMC3659725 23197860

[15] Mantovani A, Sozzani S, Locati M, Allavena P, Sica A (2002) Macrophage polarization: tumor-associated macrophages as a paradigm for polarized M2 mononuclear phagocytes. Trends Immunol 23: 549–555. 12401408 10.1016/s1471-4906(02)02302-5

[16] Martinez FO, Gordon S, Locati M, Mantovani A (2006) Transcriptional profiling of the human monocyte-to-macrophage differentiation and polarization: new molecules and patterns of gene expression. J Immunol 177: 7303–7311. 17082649 10.4049/jimmunol.177.10.7303

[17] Mosser DM, Zhang X (2008) Activation of murine macrophages. Curr Protoc Immunol Chapter 14: Unit 14 12. 10.1002/0471142735.im1402s83 PMC2822273 19016446

[18] Mosser DM, Edwards JP (2008) Exploring the full spectrum of macrophage activation. Nat Rev Immunol 8: 958–969. 19029990 10.1038/nri2448 PMC2724991

[19] Mantovani A, Sica A, Locati M (2007) New vistas on macrophage differentiation and activation. Eur J Immunol 37: 14–16. 17183610 10.1002/eji.200636910

[20] Coffelt SB, Tomchuck SL, Zwezdaryk KJ, Danka ES, Scandurro AB (2009) Leucine leucine-37 uses formyl peptide receptor-like 1 to activate signal transduction pathways, stimulate oncogenic gene expression, and enhance the invasiveness of ovarian cancer cells. Mol Cancer Res 7: 907–915. 19491199 10.1158/1541-7786.MCR-08-0326 PMC2755540

[21] Roby KF, Taylor CC, Sweetwood JP, Cheng Y, Pace JL, et al (2000) Development of a syngeneic mouse model for events related to ovarian cancer. Carcinogenesis 21: 585–591. 10753190 10.1093/carcin/21.4.585

[22] Ribatti D, Nico B, Finato N, Crivellato E (2011) Tryptase-positive mast cells and CD8-positive T cells in human endometrial cancer. Pathol Int 61: 442–444. 21707849 10.1111/j.1440-1827.2011.02680.x

[23] Murdoch C, Muthana M, Coffelt SB, Lewis CE (2008) The role of myeloid cells in the promotion of tumour angiogenesis. Nat Rev Cancer 8: 618–631. 18633355 10.1038/nrc2444

[24] Navi D, Saegusa J, Liu FT (2007) Mast cells and immunological skin diseases. Clin Rev Allergy Immunol 33: 144–155. 18094953 10.1007/s12016-007-0029-4

[25] Brown JM, Nemeth K, Kushnir-Sukhov NM, Metcalfe DD, Mezey E (2011) Bone marrow stromal cells inhibit mast cell function via a COX2-dependent mechanism. Clin Exp Allergy 41: 526–534. 21255158 10.1111/j.1365-2222.2010.03685.x PMC3078050

[26] Bianchi G, Borgonovo G, Pistoia V, Raffaghello L (2011) Immunosuppressive cells and tumour microenvironment: focus on mesenchymal stem cells and myeloid derived suppressor cells. Histol Histopathol 26: 941–951. 21630223 10.14670/HH-26.941

[27] Djouad F, Plence P, Bony C, Tropel P, Apparailly F, et al (2003) Immunosuppressive effect of mesenchymal stem cells favors tumor growth in allogeneic animals. Blood 102: 3837–3844. 12881305 10.1182/blood-2003-04-1193

[28] Khakoo AY, Pati S, Anderson SA, Reid W, Elshal MF, et al (2006) Human mesenchymal stem cells exert potent antitumorigenic effects in a model of Kaposi's sarcoma. J Exp Med 203: 1235–1247. 16636132 10.1084/jem.20051921 PMC2121206

[29] Shinagawa K, Kitadai Y, Tanaka M, Sumida T, Kodama M, et al (2010) Mesenchymal stem cells enhance growth and metastasis of colon cancer. Int J Cancer 127: 2323–2333. 20473928 10.1002/ijc.25440

[30] Gialeli C, Theocharis AD, Karamanos NK (2010) Roles of matrix metalloproteinases in cancer progression and their pharmacological targeting. FEBS J 278: 16–27. 21087457 10.1111/j.1742-4658.2010.07919.x

[31] Cubillos-Ruiz JR, Rutkowski M, Conejo-Garcia JR (2010) Blocking ovarian cancer progression by targeting tumor microenvironmental leukocytes. Cell Cycle 9: 260–268. 20023378 10.4161/cc.9.2.10430 PMC3056209

[32] Nesbeth Y, Scarlett U, Cubillos-Ruiz J, Martinez D, Engle X, et al (2009) CCL5-mediated endogenous antitumor immunity elicited by adoptively transferred lymphocytes and dendritic cell depletion. Cancer Res 69: 6331–6338. 19602595 10.1158/0008-5472.CAN-08-4329 PMC2755640

[33] Prockop DJ, Youn Oh J (2011) Mesenchymal Stem/Stromal Cells (MSCs): Role as Guardians of Inflammation. Mol Ther. 10.1038/mt.2011.211 PMC3255583 22008910

[34] Singer NG, Caplan AI (2011) Mesenchymal stem cells: mechanisms of inflammation. Annu Rev Pathol 6: 457–478. 21073342 10.1146/annurev-pathol-011110-130230

[35] Condeelis J, Pollard JW (2006) Macrophages: obligate partners for tumor cell migration, invasion, and metastasis. Cell 124: 263–266. 16439202 10.1016/j.cell.2006.01.007

[36] Baay M, Brouwer A, Pauwels P, Peeters M, Lardon F (2011) Tumor cells and tumor-associated macrophages: secreted proteins as potential targets for therapy. Clin Dev Immunol 2011: 565187. 22162712 10.1155/2011/565187 PMC3227419

[37] Sica A, Bronte V (2007) Altered macrophage differentiation and immune dysfunction in tumor development. J Clin Invest 117: 1155–1166. 17476345 10.1172/JCI31422 PMC1857267

[38] Ingman WV, Wyckoff J, Gouon-Evans V, Condeelis J, Pollard JW (2006) Macrophages promote collagen fibrillogenesis around terminal end buds of the developing mammary gland. Dev Dyn 235: 3222–3229. 17029292 10.1002/dvdy.20972

[39] Coffelt SB, Hughes R, Lewis CE (2009) Tumor-associated macrophages: Effectors of angiogenesis and tumor progression. Biochim Biophys Acta 1796: 11–18. 19269310 10.1016/j.bbcan.2009.02.004

[40] Coussens LM, Werb Z (2002) Inflammation and cancer. Nature 420: 860–867. 12490959 10.1038/nature01322 PMC2803035

[41] Fidler IJ (2003) The pathogenesis of cancer metastasis: the ‘seed and soil’ hypothesis revisited. Nat Rev Cancer 3: 453–458. 12778135 10.1038/nrc1098

[42] Rabinovich GA, Gabrilovich D, Sotomayor EM (2007) Immunosuppressive strategies that are mediated by tumor cells. Annu Rev Immunol 25: 267–296. 17134371 10.1146/annurev.immunol.25.022106.141609 PMC2895922

[43] Strausberg RL (2005) Tumor microenvironments, the immune system and cancer survival. Genome Biol 6: 211. 15774034 10.1186/gb-2005-6-3-211 PMC1088935

[44] Mason C, Brindley DA, Culme-Seymour EJ, Davie NL (2011) Cell therapy industry: billion dollar global business with unlimited potential. Regen Med 6: 265–272. 21548728 10.2217/rme.11.28

[45] Tomchuck SL, Zwezdaryk KJ, Coffelt SB, Waterman RS, Danka ES, et al (2008) Toll-like receptors on human mesenchymal stem cells drive their migration and immunomodulating responses. Stem Cells 26: 99–107. 17916800 10.1634/stemcells.2007-0563 PMC2757778

[46] Liu Y, Zeng B, Zhang Z, Zhang Y, Yang R (2008) B7-H1 on myeloid-derived suppressor cells in immune suppression by a mouse model of ovarian cancer. Clin Immunol 129: 471–481. 18790673 10.1016/j.clim.2008.07.030

[47] Ohtaki H, Ylostalo JH, Foraker JE, Robinson AP, Reger RL, et al (2008) Stem/progenitor cells from bone marrow decrease neuronal death in global ischemia by modulation of inflammatory/immune responses. Proc Natl Acad Sci U S A 105: 14638–14643. 18794523 10.1073/pnas.0803670105 PMC2567180

[48] Yang R, Cai Z, Zhang Y, Yutzy WH, Roby KF, et al (2006) CD80 in immune suppression by mouse ovarian carcinoma-associated Gr-1+CD11b+ myeloid cells. Cancer Res 66: 6807–6815. 16818658 10.1158/0008-5472.CAN-05-3755

[49] Coffelt SB, Waterman RS, Florez L, Honer zu Bentrup K, Zwezdaryk KJ, et al (2008) Ovarian cancers overexpress the antimicrobial protein hCAP-18 and its derivative LL-37 increases ovarian cancer cell proliferation and invasion. Int J Cancer 122: 1030–1039. 17960624 10.1002/ijc.23186

