# Supplementary figures and images for: Mesenchymal Stem Cell 1 (MSC1)-Based Therapy Attenuates Tumor Growth Whereas MSC2-Treatment Promotes Tumor Growth and Metastasis
Source: PLoS One. 2012 Sep 20;7(9):e45590. doi: 10.1371/journal.pone.0045590 (PMC3447765; doi:10.1371/journal.pone.0045590)

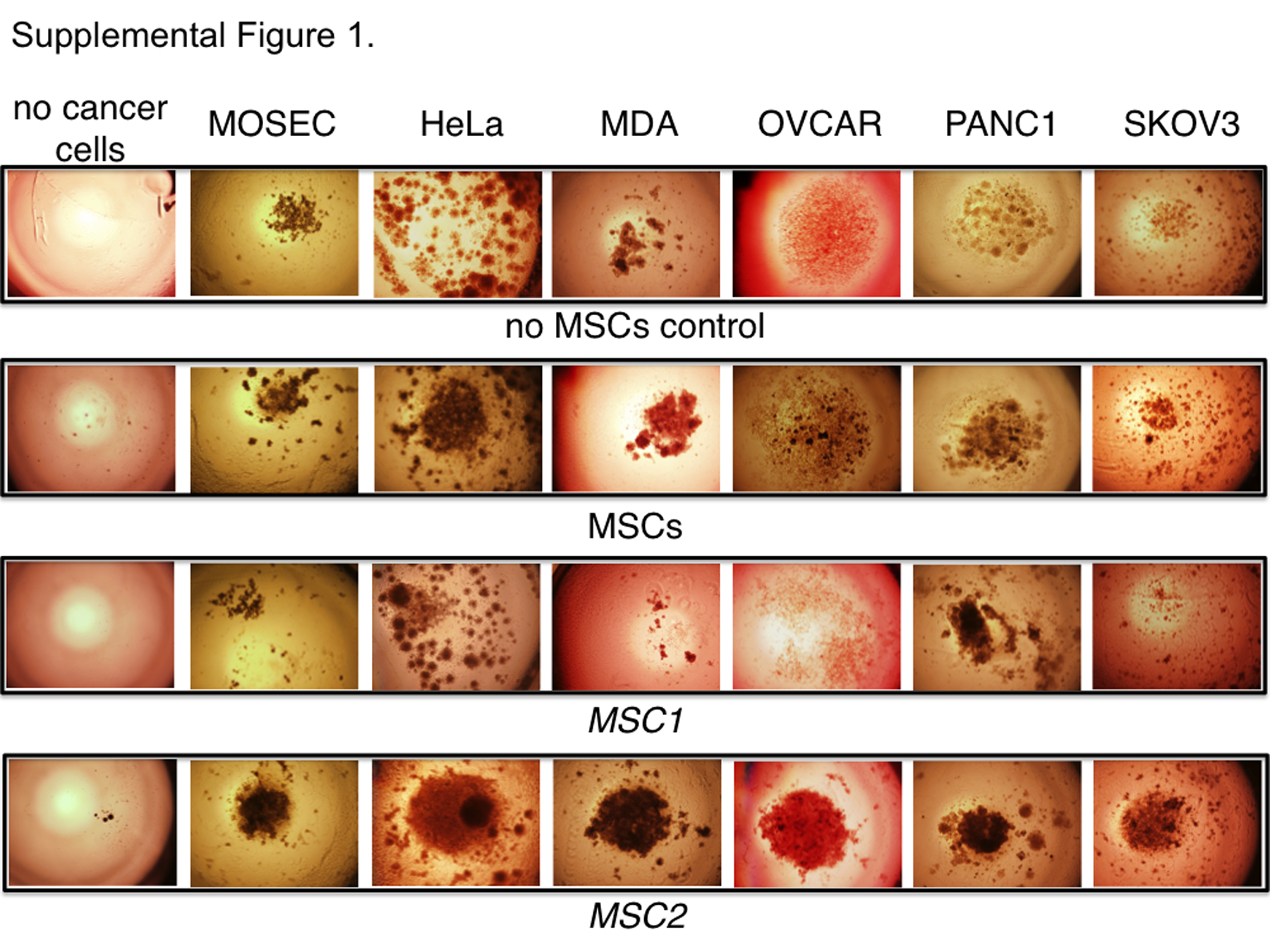

Supplement: Figure S1 — MSC1 diminish tumor growth whereas MSC2 favor tumor growth. Tumor spheroids were formed by culturing tumor cells (200 cells/well) mixed without any other cells (–) or with CellTracker green labeled MSCs, MSC1, or MSC2 (20 cells/well) at a ratio of 10 cancer cells per 1 MSC and plated over 1.5% agarose in 96-well plates in growth medium supplemented with 10% FBS as indicated in figure. Cultures were grown for 14 days at 37°C in a humidified atmosphere of 5% carbon dioxide balance air. Growth medium was changed every 3–4 days. Representative micrographs shown represent 20X magnified bright field of the 96-well plate. Cancer cell lines used are: HeLa- human cervical adenocarcinoma, PANC-1- human pancreatic adenocarcinoma, OVCAR-human ovarian adenocarcinoma, SKOV3-human ovarian adenocarcinoma, and MOSEC-murine ovarian surface epithelium carcinoma cells. (TIF) [file pone.0045590.s001.tif]

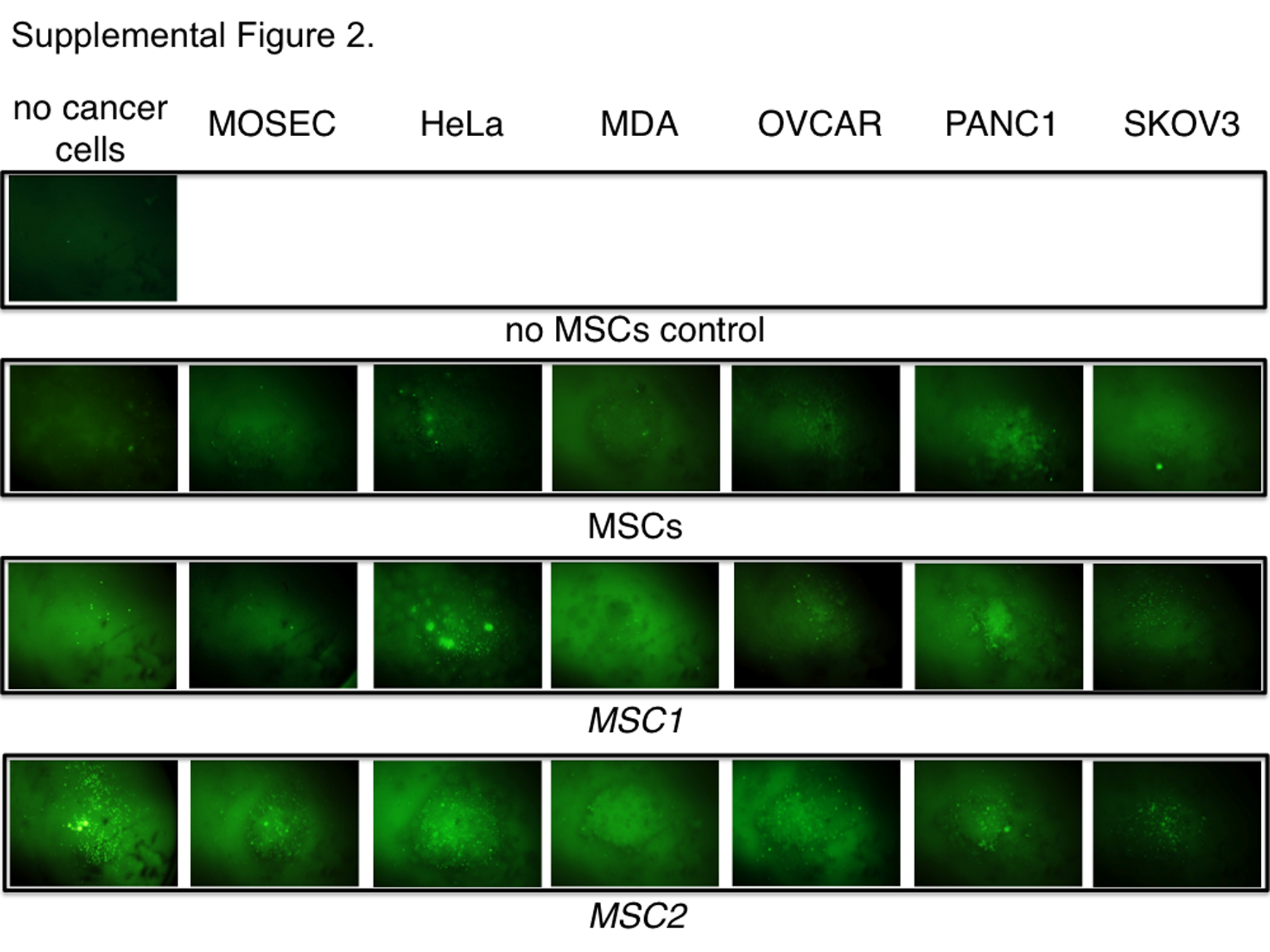

Supplement: Figure S2 — MSC1 diminish tumor growth whereas MSC2 favor tumor growth. Fluorescence micrographs corresponding to those bright field micrographs presented in Figure S1. CellTracker green labeled MSCs, MSC1, or MSC2 appear as the brighter spots in the images. It appears that the cells distribute throughout the tumor spheroids–whose shadows are visible in these fluorescence micrographs. (TIF) [file pone.0045590.s002.tif]

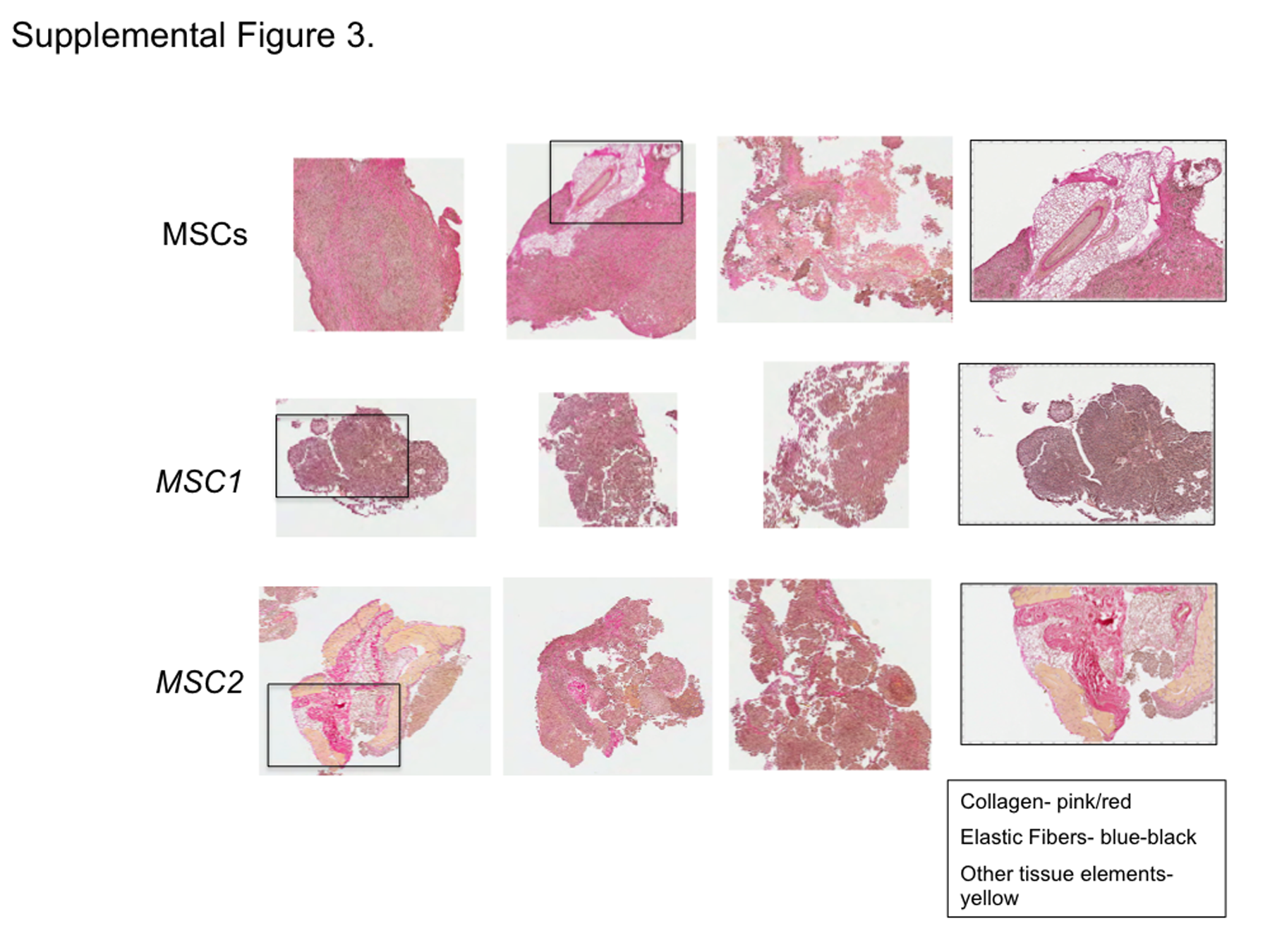

Supplement: Figure S3 — MSC1 -treated tumor samples have diminished levels of collagen within the TME compared to MSC2 - and MSC-treated tumor groups. MOSEC tumors were established in C57BL/6 mice for 4 weeks. MSCs, MSC1, or MSC2 (1×10^6^ in 0.5 mL HBSS) were infused IP and the mice were harvested after 65 days. Tumors were excised, fixed, and cut into 5 µM sections by standard methods [7]. Sections were processed for Verhoeff-Van Gieson (VVG) elastic fiber/collagen staining (www.ihcworld.com). Representative micrographs of several MSC-treated tumor sections are included from images obtained from the Aperio ScanScope (40X, Aperio, Vista, CA). The expected color for each tissue element is described in the inset on the lower right hand side. 80X images are included in boxed insets. Data are representative of three independent experiments with at least 6 mice per treatment group. (TIF) [file pone.0045590.s003.tif]

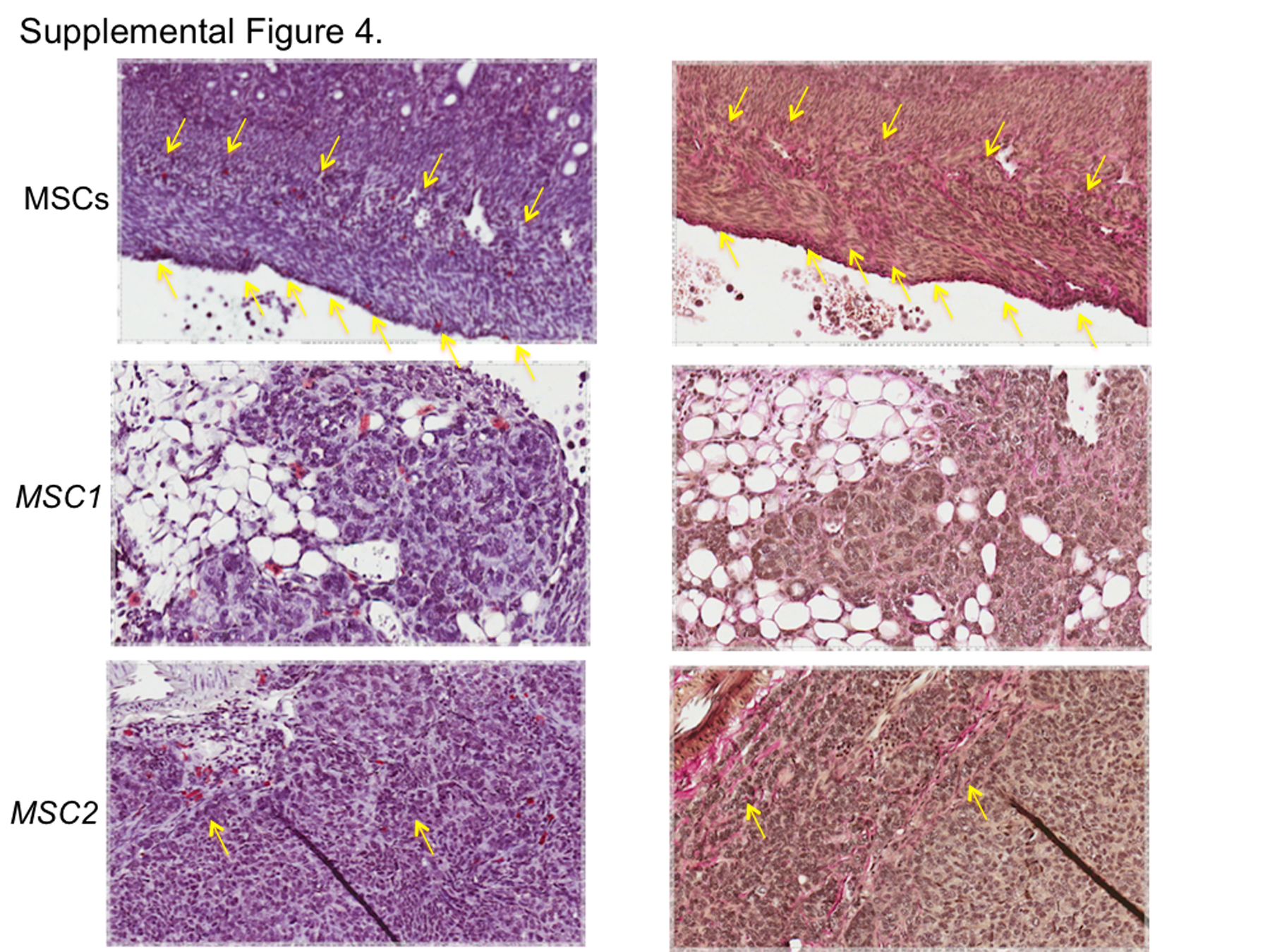

Supplement: Figure S4 — Co-localization of tumor associated mast cells with collagen. MOSEC tumors were established in C57BL/6 mice for 4 weeks. MSCs, MSC1, or MSC2 (1×10^6^ in 0.5 mL HBSS) were infused IP and the mice were harvested after 65 days. Tumors were excised, fixed, and cut into 5 µM sections by standard methods [7]. Sections were processed for Verhoeff-Van Gieson (VVG) elastic fiber/collagen staining (left panels) or for safranin O proteoglycan staining (right panels, www.ihcworld.com). Representative micrographs of several MSC-treated tumor sections are included from images obtained from the Aperio ScanScope (40X, Aperio, Vista, CA). Yellow arrows indicate comparable sections among the tumor tissue sections. Data are representative of three independent experiments with at least 6 mice per treatment group. (TIF) [file pone.0045590.s004.tif]
